# Supplementary material for: Function and phylogeny support the independent evolution of an ASIC-like Deg/ENaC channel in the Placozoa
Source: Commun Biol. 2023 Sep 18;6:951. doi: 10.1038/s42003-023-05312-0 (PMC10507113; doi:10.1038/s42003-023-05312-0)
Supplement: Supplementary file 2 — Supplementary Materials [file 42003_2023_5312_MOESM2_ESM.pdf]

# Function and phylogeny support the independent evolution of an ASIC-like Deg/ENaC channel in the Placozoa

Wassim Elkhatib[1,2], Luis Yanez-Guerra[3], Tatiana D. Mayorova[4], Mark A. Currie[1,2], Anhadvir Singh[1,2], Maria Perera[1,2], Julia Gauberg[1,2], and Adriano Senatore[1,2*]

[1]Department of Biology, University of Toronto Mississauga, 3359 Mississauga Road, Mississauga, ON L5L 1C6, Canada.

[2]Department of Cell and Systems Biology, University of Toronto, 25 Harbord Street, Toronto, ON M5S 3G5, Canada.

[3]Living Systems Institute, University of Exeter, Stocker Road, Exeter, EX4 4QD, England.

[4]NINDS, National Institutes of Health, Bethesda Maryland, 20892 USA.

*Corresponding author: Adriano Senatore

E-mail: adriano.senatore@utoronto.ca

Tel. (905) 569-4322

Supplementary Figure 1 - Page 2
Supplementary Figure 2 - Page 3
Supplementary Figure 3 - Page 3
Supplementary Figure 4 - Page 4
Supplementary Figure 5 - Page 4

Supplementary Table 1 - Page 5
Supplementary Table 2 - Page 5

Supplementary Data Legend - Page 6

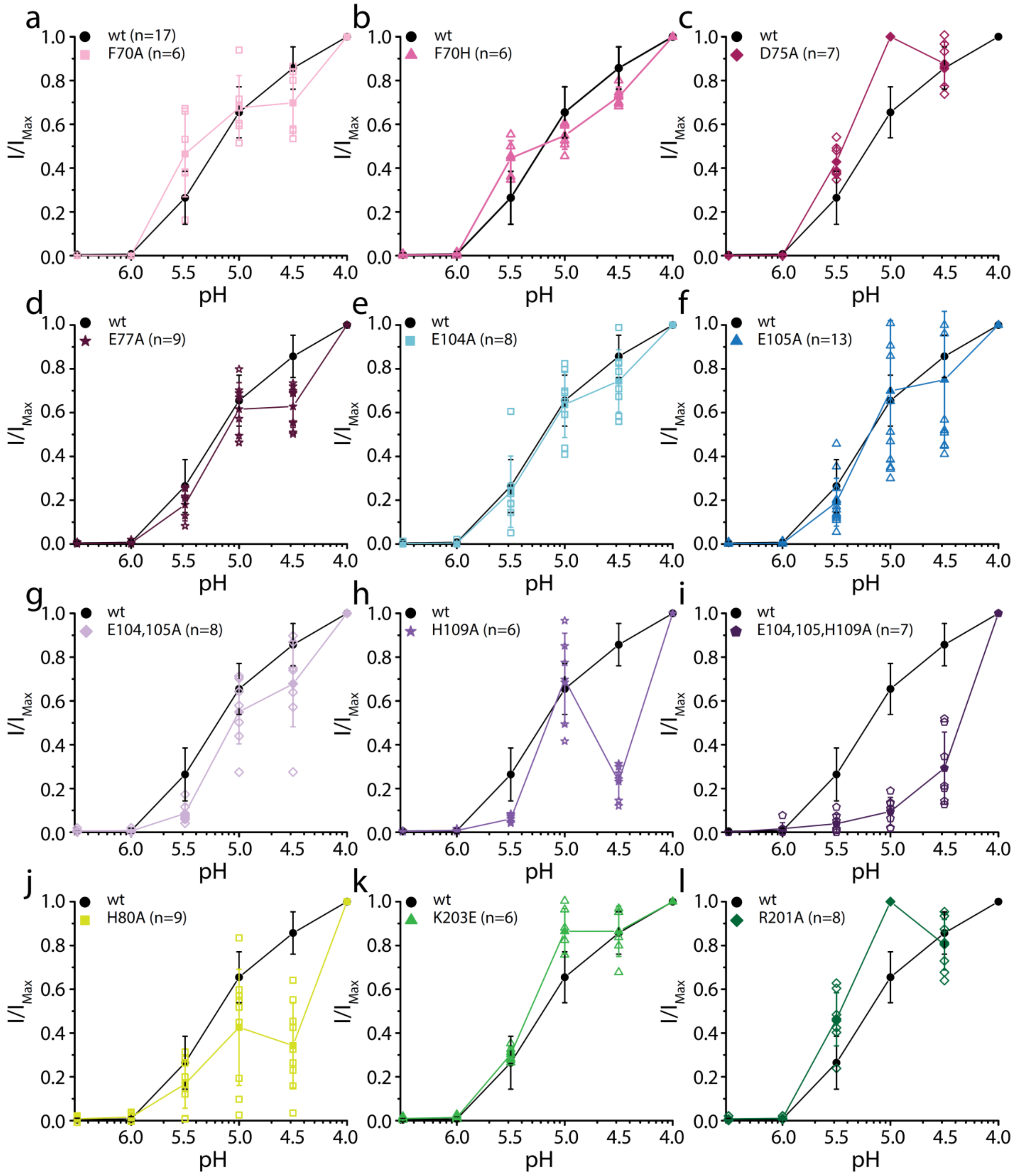


**Supplementary Figure 1.** Average pH dose response curves for *Tad*NaC2 channel variants bearing mutations in the wrist region (*a* to *d*), the knuckle region (*e* to *i*), and palm region (*j* to *l*).

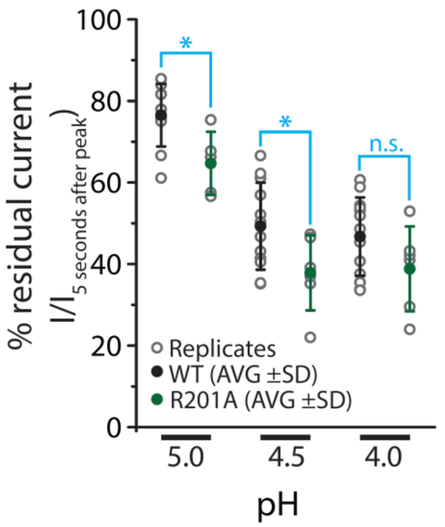


**Supplementary Figure 2.** Plot of percent residual current at 5 seconds after peak for the *Tad*NaC2 wildtype and R201A channel variants at different pH. The asterisks denote statistically significant p values $\leq 0.05$ for two-sample t-tests.

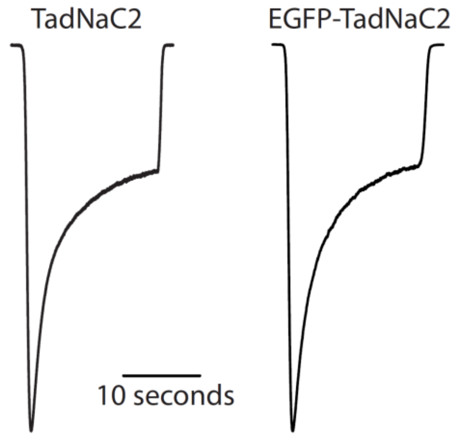


**Supplementary Figure 3.** Sample traces recordings of untagged and EGFP-tagged *Tad*NaC2 channels in response to activation by a pH 4.5 solution.

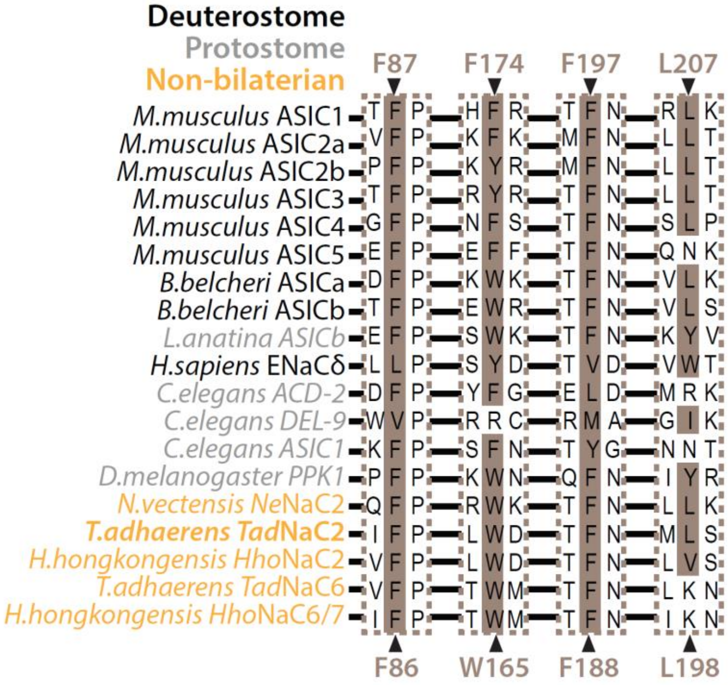


**Supplementary Figure 4.** Protein sequence alignment reveals conserved aromatic residues that contribute to the hydrophobic hub in ASIC channels.

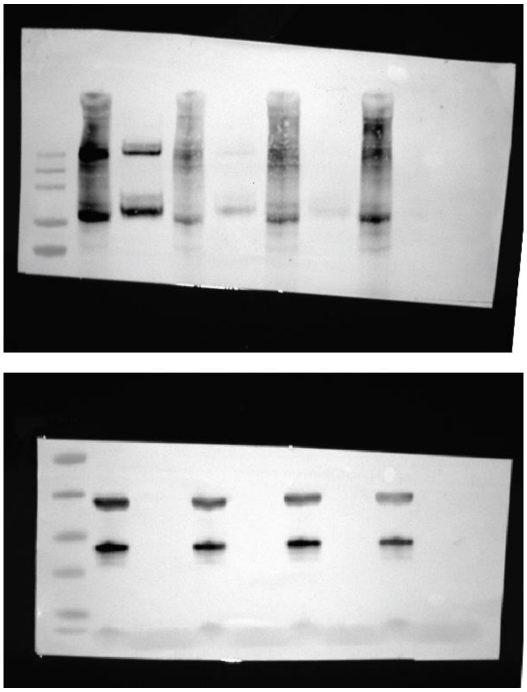

**Supplementary Figure 5.** Raw uncropped images of Western blots presented in Fig. 8.

**Supplementary Table 1.** BUSCO completeness scores and accession details for the various genetic databases used in this study.

| Clade | Clade | Phylum | Subphylum | Species | Source | Accession/link | Completeness | Singles | Doubles | Fragmented | Missing | BUSCOS |
|---|---|---|---|---|---|---|---|---|---|---|---|---|
| Ambulacraria | | Echinodermata | | *Acanthaster planci* | | https://marinegenomics.oist.jp/cots/viewer/download?project_id=46 | 91.4 | 91.4 | 0 | 5.9 | 2.7 | n:255 |
| Ambulacraria | | Echinodermata | | *Strongylocentrotus purpuratus* | UniProt | UP000007110 | 86.3 | 80 | 6.3 | 11 | 2.7 | n:255 |
| Ambulacraria | | Hemichordata | | *Saccoglossus kowalevskii* | | https://groups.oist.jp/molgenu/hemichordate-genomes | 94.1 | 91.4 | 2.7 | 3.5 | 2.4 | n:255 |
| Ambulacraria | | Hemichordata | | *Ptychodera flava* | | https://groups.oist.jp/molgenu/hemichordate-genomes | 53 | 51.4 | 1.6 | 25.1 | 21.9 | n:255 |
| Deuterostomia | | Chordata | Cephalochordata | *Branchiostoma belcheri* | | http://genome.bucm.edu.cn/lancelet/download_data.php | 96.9 | 85.1 | 11.8 | 2.4 | 0.7 | n:255 |
| Deuterostomia | | Chordata | Urochordata | *Ciona intestinalis* | UniProt | UP000008144 | 85.5 | 85.5 | 0 | 7.1 | 7.4 | n:255 |
| Deuterostomia | | Chordata | Craniata | *Gallus gallus* | UniProt | UP000000539 | 96.1 | 86.3 | 9.8 | 2 | 1.9 | n:255 |
| Deuterostomia | | Chordata | Craniata | *Homo sapiens* | UniProt | UP000005640 | 100 | 97.6 | 2.4 | 0 | 0 | n:255 |
| Deuterostomia | | Chordata | Craniata | *Rattus norvegicus* | UniProt | UP000002494 | 100 | 98 | 2 | 0 | 0 | n:255 |
| Deuterostomia | | Chordata | Craniata | *Petromyzon marinus* | UniProt | UP000245300 | 54.5 | 52.9 | 1.6 | 9.8 | 35.7 | n:255 |
| | | Cnidaria | Cubozoa | *Alatina Alata* | https://bitbucket.org/caseywdunn/cnidaria2014-assemblies/src/master/Cnidaria_Transcriptomes/ | | 96.5 | 76.5 | 20 | 2.4 | 1.1 | n:255 |
| | | Cnidaria | Hydrozoa | *Hydra vulgaris* | https://research.nhgri.nih.gov/hydra/ | | 86.6 | 82.7 | 3.9 | 10.2 | 3.2 | n:255 |
| | | Cnidaria | Hexacoralia | *Nematostella vectensis* | http://metazoa.ensembl.org/species.html | | 99.2 | 92.5 | 6.7 | 0.8 | 0 | n:255 |
| | | Cnidaria | Polypodium | *Polypodium hydriforme* | https://www.ncbi.nlm.nih.gov/nuccore/671211599 | | 78.4 | 13.7 | 64.7 | 14.5 | 7.1 | n:255 |
| | | Cnidaria | Scyphozoa | *Rhopilema_esculentum* | https://academic.oup.com/gigascience/article/9/4/giaa036/5823175 | | 98.1 | 62.4 | 35.7 | 1.2 | 0.7 | n:255 |
| | | Ctenophora | Lobata | *Mnemiopsis leidyi* | https://research.nhgri.nih.gov/mnemiopsis/download/download.cgi?dl=transcript | Trinity assembly | 89.8 | 83.5 | 6.3 | 5.1 | 5.1 | n:255 |
| | | Ctenophora | Cydippida | *Pleurobrachia_bachei* | https://neurobase.rc.ufl.edu/pleurobrachia/download | | 79.6 | 30.2 | 49.4 | 13.7 | 6.7 | n:255 |
| | | Ctenophora | Cydippida | *Pukia_falcata* | https://neurobase.rc.ufl.edu/ | | 71 | 35.3 | 35.7 | 20.8 | 8.2 | n:255 |
| | | Ctenophora | Cydippida | *Hormiphora californiensis* | JGI portal (Project accession: PRJNA281977, runs accession: SRR1992642) | Trinity assembly | 94.9 | 39.6 | 55.3 | 1.6 | 3.5 | 255 |
| Protostomia | Ecdysozoa | Arthropoda | | *Centruroides sculpturatus* | PRJNA281977, runs accession: SRR1992642 | https://www.ncbi.nlm.nih.gov/genome/?term=centruroides+sculpturatus | 89.1 | 67.5 | 21.6 | 9 | 1.9 | n:255 |
| Protostomia | Ecdysozoa | Arthropoda | | *Drosophila melanogaster* | UniProt | UP000000803 | 100 | 99.6 | 0.4 | 0 | 0 | n:255 |
| Protostomia | Ecdysozoa | Arthropoda | | *Tribolium castaneum* | UniProt | UP000007266 | 97.3 | 96.9 | 0.4 | 2 | 0.7 | n:255 |
| Protostomia | Ecdysozoa | Priapulida | | *Priapulus caudatus* | NCBI | PRJNA20497 | 89 | 73.3 | 15.7 | 7.8 | 3.2 | n:255 |
| Protostomia | Ecdysozoa | Tardigrada | | *Ramazzottius varieornatus* | UniProt | UP000186922 | 92.5 | 88.2 | 4.3 | 3.1 | 4.4 | 225 |
| Protostomia | Ecdysozoa | Tardigrada | | *Hypsibius dujardini* | UniProt | UP000192578 | 76.9 | 74.9 | 2 | 4.7 | 18.4 | 225 |
| Protostomia | Ecdysozoa | Nematoda | | *Trichinella spiralis* | UniProt | UP000054776 | 87.1 | 85.5 | 1.6 | 2 | 10.9 | 255 |
| Protostomia | Ecdysozoa | Nematoda | | *Caenorhabditis elegans* | UniProt | UP000001940 | 97.7 | 96.9 | 0.8 | 0.8 | 1.5 | 255 |
| Protostomia | Lophotrochozoa | Mollusca | | *Aplysia californica* | UniProt | UP000694888 | 98.4 | 75.3 | 23.1 | 0.8 | 0.8 | 255 |
| Protostomia | Lophotrochozoa | Mollusca | | *Octopus bimaculoides* | UniProt | UP000053454 | 87.9 | 87.5 | 0.4 | 9.4 | 2.7 | n:255 |
| Protostomia | Lophotrochozoa | Brachiopoda | | *Lingula_anatina* | https://marinegenomics.oist.jp/lan_v2/viewer/info?project_id=50 | | 94.9 | 72.2 | 22.7 | 3.9 | 1.2 | n:255 |
| Protostomia | Lophotrochozoa | Annelida | | *Owenia fusiformis* | NCBI | https://www.ncbi.nlm.nih.gov/assembly/GCA_903813345.2 | 93.7 | 84.3 | 9.4 | 2 | 4.3 | n:255 |
| Protostomia | Lophotrochozoa | Annelida | | *Platynereis dumerilii* | PdumBase | http://pdumbase.gdcb.iastate.edu/ | 90.6 | 87.5 | 3.1 | 5.5 | 3.9 | n:255 |
| Protostomia | Lophotrochozoa | Platyhelminthes | | *Schistosoma mansoni* | NCBI | UP000008854 | 95.3 | 78.4 | 16.9 | 2 | 2.7 | 255 |
| Protostomia | Lophotrochozoa | Platyhelminthes | | *Macrostomum lignano* | NCBI | UP000215902 | 91.8 | 12.2 | 79.6 | 3.1 | 5.1 | 255 |
| Protostomia | Lophotrochozoa | Phoronida | | *Phoronis australis* | NCBI | https://www.ncbi.nlm.nih.gov/assembly/GCA_002633005.1/ | 96 | 73.3 | 22.7 | 2.4 | 1.6 | 255 |
| Protostomia | Lophotrochozoa | Rotifera | | *Rotaria socialis* | UniProt | | 95.7 | 79.2 | 16.5 | 2 | 2.3 | 255 |
| | | Placozoa | Trichoplacidae | *Hoilungia hongkongensis* | | https://bitbucket.org/molpalmuc/hoilungia-genome/src/master/sequences/Hhon_TRINITY_transdecoder_proteins.fasta.gz | 92.9 | 89 | 3.9 | 2.4 | 4.7 | n:255 |
| | | Placozoa | Trichoplacidae | *Trichoplax adhaerens* | NCBI | GHJI00000000.1 | 92.9 | 92.5 | 0.4 | 4.7 | 2.4 | n:255 |
| | | Porifera | Demospongiae | *Amphimedon queenslandica* | UniProt | UP000007879 | 92.5 | 88.2 | 4.3 | 7.1 | 0.4 | n:255 |
| | | Porifera | Demospongiae | *Ephydatia muelleri* | | https://spaces.facsci.ualberta.ca/ephybase/ | 97.3 | 81.2 | 16.1 | 1.6 | 1.1 | n:255 |
| | | Porifera | Homoscleromorpha | *Oscarella carmela* | COMPAGEN | | 95.3 | 87.1 | 8.2 | 2.7 | 2 | n:255 |
| | | Porifera | Calcarea | *Sycon ciliatum* | COMPAGEN | | 97.3 | 91 | 6.3 | 2 | 0.7 | n:255 |
| Choanoflagellatea | | Salpingoecidae | | *Monosiga_brevicolis* | UniProt | UP000001357 | 78.8 | 78.8 | 0 | 7.5 | 13.7 | n:255 |
| Choanoflagellatea | | Salpingoecidae | | *Salpingoeca rosetta* | UniProt | UP000007799 | 83.1 | 83.1 | 0 | 6.7 | 10.2 | n:255 |
| | | | | | | | | | | | | |
| Filasterea | | | | *Capsaspora owczarzaki* | UniProt | UP000008743 | 93.7 | 92.9 | 0.8 | 2.4 | 3.9 | n:255 |
| Filasterea-related "pluriformea" | | position debated | | *Tunicaraptor unikontum* | NCBI | PRJNA638967 | 74.1 | 64.7 | 9.4 | 16.5 | 9.4 | n:255 |
| | | | | | | | | | | | | |
| Alveolata | | Symbiodinium | | Symbyodinium sp_KB8 | NCBI | https://www.ncbi.nlm.nih.gov/assembly/GCA_905221625.1/#/def | 63.4 | 44.3 | 20 | 12.2 | 23.5 | n:255 |
| Stramenopiles | | Cafeteriaceae | | Cafeteria roenbergensis | NCBI | https://www.ncbi.nlm.nih.gov/bioproject/PRJNA552725/ | 67.1 | 65.9 | 1.2 | 9 | 23.9 | 255 |

**Supplementary Table 2.** Primers used for cloning *Tad*NaC2 constructs.

| Primer Name | DNA Sequence (5' to 3') |
|---|---|
| TadNaC2_F1 | CTTTCGACACTGCTGTACAGCTC |
| TadNaC2_F2 | ATTATACTCGAGGCCGCCACCATGGATACTCATCCTTGGCAAAGGCC |
| TadNaC2_R1 | GATGATAATCTAAACATCGAACTACGTAGC |
| TadNaC2_R2 | ATTATAGGATCCAACCTAGTCAATACTGGATGGACTAATTCG |
| TadNaC10_F1 | ACGACACTGAAACAGCTTTCGCG |
| TadNaC10_F2 | ATTATACTCGAGGCCGCCACCATGCCAACCAAGCAGTTAAAGGTTATGAG |
| TadNaC10_R1 | GTTGATAATGAAAGTATCGGTCTTGCGACG |
| TadNaC10_R2 | ATTATAGGATCCACTTTATGCAAGTCCAGAAGCTTGCAACTG |
| TadNaC2_F70A_F | TTGCAGTATGTTTTATCCGCCCCAACGAATATTGATATC |
| TadNaC2_F70A_R | GATATCAATATTCGTTGGGGCGGATAAAACATACTGCAA |
| TadNaC2_F70H_F | TTGCAGTATGTTTTATCCCACCCAACGAATATTGATATC |
| TadNaC2_F70H_R | GATATCAATATTCGTTGGGTGGGATAAAACATACTGCAA |
| TadNaC2_D75A_F | CCTTTCCAACGAATATTGCCATCGAAATTATACACCAAG |
| TadNaC2_D75A_R | CTTGGTGTATAATTTCGATGGCAATATTCGTTGGAAAGG |
| TadNaC2_E77A_F | CCAACGAATATTGATATCGCCATTATACACCAAGATAGC |
| TadNaC2_E77A_R | GCTATCTTGGTGTATAATGGCGATATCAATATTCGTTGG |
| TadNaC2_H80A_F | ATTGATATCGAAATTATAGCCCAAGATAGCTTAATATTC |
| TadNaC2_H80A_R | GAATATTAAGCTATCTTGGGCTATAATTTCGATATCAAT |
| TadNaC2_E104A_F | CCAAAACAGCATTATCGGCCGAAGATAATCGACACTTGC |
| TadNaC2_E104A_R | GCAAGTGTCGATTATCTTCGGCCGATAATGCTGTTTTGG |
| TadNaC2_E105A_F | CCAAAACAGCATTATCGGAAGCCGATAATCGACACTTGC |
| TadNaC2_E105A_R | GCAAGTGTCGATTATCGGCTTCCGATAATGCTGTTTTGG |
| TadNaC2_E104-5A_F | CCAAAACAGCATTATCGGCCGCCGATAATCGACACTTGC |
| TadNaC2_E104-5A_R | GCAAGTGTCGATTATCGGCGGCCGATAATGCTGTTTTGG |
| TadNaC2_H109A_F | TCGGAAGAAGATAATCGAGCCTTGCAGACATTTCTTAGA |
| TadNaC2_H109A_R | TCTAAGAAATGTCTGCAAGGCTCGATTATCTTCTTCCGA |
| TadNaC2E104-5-9AF | CCAAAACAGCATTATCGGCCGCCGATAATCGAGCCTTGCAGACATTTCTTAGA |
| TadNaC2E104-5-9AR | TCTAAGAAATGTCTGCAAGGCTCGATTATCGGCGGCCGATAATGCTGTTTTGG |
| TadNaC2_R201A_F | GAAGGTATGTTATCTCAGGCCGGGAAAGGCTCTGCACAC |
| TadNaC2_R201A_R | GTGTGCAGAGCCTTTCCCGGCCTGAGATAACATACCTTC |
| TadNaC2_K203E_F | ATGTTATCTCAGCGTGGGGAGGGCTCTGCACACGGACTACG |
| TadNaC2_K203E_R | CGTAGTCCGTGTGCAGAGCCCTCCCCACGCTGAGATAACAT |
| TadNaC2_K203Del_F | ATGTTATCTCAGCGTGGGGGCTCTGCACACGGACTACG |
| TadNaC2_K203Del_R | CGTAGTCCGTGTGCAGAGCCCCCACGCTGAGATAACAT |

## Supplementary Data Legend

**Supplementary Data 1.** FASTA query file, including sequences and accession numbers used in BLAST to identify DEG/ENaCs.

**Supplementary Data 2.** FASTA file containing protein sequences of identified Deg/ENaC channels used to generate the cluster map.

**Supplementary Data 3.** CLANS cluster map file generated using a P value cut-off of 1E-10.

**Supplementary Data 4.** CLANS cluster map file generated using a P value cut-off of 1E-20.

**Supplementary Data 5.** CLANS cluster map file generated using a P value cut-off of 1E-30.

**Supplementary Data 6.** CLANS cluster map file generated using a P value cut-off of 1E-40.

**Supplementary Data 7.** CLANS cluster map file generated using a P value cut-off of 1E-50.

**Supplementary Data 8.** FASTA file containing raw and refined protein sequences used to generate the tree presented in Fig. 2.

**Supplementary Data 9.** Nexus file of the phylogenetic tree presented in Figure 2.

**Supplementary Data 10.** FASTA file containing MAFFT-aligned protein sequences used to generate the tree presented in Figure 2.

**Supplementary Data 11.** FASTA file containing aligned and trimmed protein sequences used to generate the tree presented in Figure 2.

**Supplementary Data 12.** Excel sheet bearing source data for plots presented in Figures 3, 4, 5, 7, and Supplementary Figure 2.
