# Supplementary material for: Function and phylogeny support the independent evolution of an ASIC-like Deg/ENaC channel in the Placozoa
Source: Commun Biol. 2023 Sep 18;6:951. doi: 10.1038/s42003-023-05312-0 (PMC10507113; doi:10.1038/s42003-023-05312-0)
Supplement: Supplementary file 3 — Description of Additional Supplementary Data [file 42003_2023_5312_MOESM3_ESM.docx]

**Description of Additional Supplementary Files**

**File name:** Supplementary Data 1

**Description:** FASTA query file, including sequences and accession numbers used in BLAST to identify DEG/ENaCs.

**File name:** Supplementary Data 2

**Description:** FASTA file containing protein sequences of identified Deg/ENaC channels used to generate the cluster map.

**File name:** Supplementary Data 3

**Description:** CLANS cluster map file generated using a P value cut-off of 1E-10.

**File name:** Supplementary Data 4

**Description:** CLANS cluster map file generated using a P value cut-off of 1E-20.

**File name:** Supplementary Data 5

**Description:** CLANS cluster map file generated using a P value cut-off of 1E-30.

**File name:** Supplementary Data 6

**Description:** CLANS cluster map file generated using a P value cut-off of 1E-40.

**File name:** Supplementary Data 7

**Description:** CLANS cluster map file generated using a P value cut-off of 1E-50.

**File name:** Supplementary Data 8

**Description:** FASTA file containing raw and refined protein sequences used to generate the tree presented in Fig. 2.

**File name:** Supplementary Data 9

**Description:** Nexus file of the phylogenetic tree presented in Figure 2.

**File name:** Supplementary Data 10

**Description:** FASTA file containing MAFFT-aligned protein sequences used to generate the tree presented in Figure 2.

**File name:** Supplementary Data 11

**Description:** FASTA file containing aligned and trimmed protein sequences used to generate the tree presented in Figure 2.

**File name:** Supplementary Data 12

**Description:** Excel sheet bearing source data for plots presented in Figures 3, 4, 5, 7, and Supplementary Figure 2.
