# Supplementary material for: Function and phylogeny support the independent evolution of an ASIC-like Deg/ENaC channel in the Placozoa
Source: Commun Biol. 2023 Sep 18;6:951. doi: 10.1038/s42003-023-05312-0 (PMC10507113; doi:10.1038/s42003-023-05312-0)
Supplement: Supplementary file 16 — Reporting Summary [file 42003_2023_5312_MOESM16_ESM.pdf]

nature portfolio

Corresponding author(s): Adriano Senatore

Last updated by author(s): Aug 22, 2023

Reporting Summary

Nature Portfolio wishes to improve the reproducibility of the work that we publish. This form provides structure for consistency and transparency in reporting. For further information on Nature Portfolio policies, see our Editorial Policies and the Editorial Policy Checklist.

Statistics

For all statistical analyses, confirm that the following items are present in the figure legend, table legend, main text, or Methods section.

n/a	Confirmed	
☐	☒	The exact sample size (*n*) for each experimental group/condition, given as a discrete number and unit of measurement
☐	☒	A statement on whether measurements were taken from distinct samples or whether the same sample was measured repeatedly
☐	☒	The statistical test(s) used AND whether they are one- or two-sided<br>*Only common tests should be described solely by name; describe more complex techniques in the Methods section.*
☒	☐	A description of all covariates tested
☐	☒	A description of any assumptions or corrections, such as tests of normality and adjustment for multiple comparisons
☐	☒	A full description of the statistical parameters including central tendency (e.g. means) or other basic estimates (e.g. regression coefficient) AND variation (e.g. standard deviation) or associated estimates of uncertainty (e.g. confidence intervals)
☐	☒	For null hypothesis testing, the test statistic (e.g. *F*, *t*, *r*) with confidence intervals, effect sizes, degrees of freedom and *P* value noted<br>*Give P values as exact values whenever suitable.*
☐	☒	For Bayesian analysis, information on the choice of priors and Markov chain Monte Carlo settings
☒	☐	For hierarchical and complex designs, identification of the appropriate level for tests and full reporting of outcomes
☒	☐	Estimates of effect sizes (e.g. Cohen's *d*, Pearson's *r*), indicating how they were calculated

Our web collection on statistics for biologists contains articles on many of the points above.

Software and code

Policy information about availability of computer code

Data collection

The whole-cell patch clamp electrophysiology data was collected using pCLamp 11 Software.

Data analysis

pClamp 11 Software was used to analyze the electrophysiological recordings of the ion channels.
Molecular structures were analyzed (and visualized) using the UCSD ChimeraX software.
Behavioural analysis of animal surface area and movement speed was done using ImageJ thresholding and the Trackmate plugin.
ImageJ was also used to analyze the integrated densities of fluorescence of transfected CHO-K1 cells.

The CLans Analysis was done using CLANS and other programs listed below:
1) Muscle for protein sequence alignments.
2) trimAl for trimming protein alignments.
3) HMMR3 for identification of homologous sequences.
4) CLANS (Java software) for analyzing protein sequences through pairwise similarities.

Building the phylogenetic tree involved using multiple softwares:
1) Phobius for confirming the presence of transmembrane domains in candidate sequences.
2) MAFFT (version 7) to align protein sequences.
3) trimAl for automated protein alignment trimming .
4) IQ-Tree for inference of a maximum likelihood phylogenetic tree and ultrafast bootstrap analysis.
5) Bootstrap method for determining node support values.

Single protein subunit structure prediction was done using AlphaFold2, and trimeric structures were derived from Phyre2 models, followed by energy minimization and geometry optimizations using the Chiron and Phenix algorithms.

For manuscripts utilizing custom algorithms or software that are central to the research but not yet described in published literature, software must be made available to editors and reviewers. We strongly encourage code deposition in a community repository (e.g. GitHub). See the Nature Portfolio guidelines for submitting code & software for further information.

Data

Policy information about availability of data

All manuscripts must include a data availability statement. This statement should provide the following information, where applicable:

- Accession codes, unique identifiers, or web links for publicly available datasets
- A description of any restrictions on data availability
- For clinical datasets or third party data, please ensure that the statement adheres to our policy

All data is available in the main text, supplementary figures (Supplementary Data 1, Supplementary Data 2, Supplementary Data 3, Supplementary Data 4, Supplementary Data 5), and Supplementary Tables (Supplementary Table 1, Supplementary Table 2). Other data can be requested from the corresponding authors upon request.

Human research participants

Policy information about studies involving human research participants and Sex and Gender in Research.

Reporting on sex and gender	N/A
Population characteristics	N/A
Recruitment	N/A
Ethics oversight	N/A

Note that full information on the approval of the study protocol must also be provided in the manuscript.

Field-specific reporting

Please select the one below that is the best fit for your research. If you are not sure, read the appropriate sections before making your selection.

☒ Life sciences ☐ Behavioural & social sciences ☐ Ecological, evolutionary & environmental sciences

For a reference copy of the document with all sections, see nature.com/documents/nr-reporting-summary-flat.pdf

Life sciences study design

All studies must disclose on these points even when the disclosure is negative.

Sample size

Electrophysiology:
No statistical methods were used to determine the sample size. In all experiments we sought to exceed the minimum required sample sizes based on our own experience recording the ion channels and their reproducible biological responses.

Protein expression analysis and western blotting:
No statistical methods were used to determine the sample size. The sample size was selected based on reproducibility of the results through multiple repetitions of experiments.

Data exclusions

Electrophysiology data:
Data was excluded if the recorded traces showed an obvious defect, for example caused by losing adhesion of the recording electrode with the cell membrane during a patch recording, or if the perfusion system became clogged and failed to deliver appropriate solutions. Recorded cells that died or showed strong decay in current amplitudes were also excluded.

Protein expression analysis:
No data was excluded from this analysis.

Replication

Electrophysiology data:
Each of the in vitro expressed ion channels and mutants were recorded over multiple days (i.e., independent transfections). The recording solutions used in the perfusion system were always applied in the same order, but their placement in the different lines of the perfusion system was changed to rule out potential differences in flow rate.

Randomization	Electrophysiology experiments:<br>CHO-K1 cells were all transfected using consistent protocols, and cells expressing the reporter fluorescent protein were randomly selected for patch clamp recording.
Blinding	No blinding was performed on any of the mentioned experiments.

Reporting for specific materials, systems and methods

We require information from authors about some types of materials, experimental systems and methods used in many studies. Here, indicate whether each material, system or method listed is relevant to your study. If you are not sure if a list item applies to your research, read the appropriate section before selecting a response.

Materials & experimental systems

n/a	Involved in the study
☐	☒ Antibodies
☐	☒ Eukaryotic cell lines
☒	☐ Palaeontology and archaeology
☒	☐ Animals and other organisms
☒	☐ Clinical data
☒	☐ Dual use research of concern

Methods

n/a	Involved in the study
☒	☐ ChIP-seq
☒	☐ Flow cytometry
☒	☐ MRI-based neuroimaging

Antibodies

Antibodies used	i) rabbit monoclonal anti-EGFP antibody, ii) rabbit monoclonal anti-GAPDH antibody, and iii) anti-rabbit secondary antibodies conjugated to horseradish peroxidase (all from Cell Signaling Technology, Massachusetts USA).
Validation	The rabbit monoclonal anti-EGFP and rabbit monoclonal anti-GAPDH antibodies were validated by Cell Signaling through western blot analysis on HCC827 cells. The anti-rabbit secondary antibodies conjugated to horseradish peroxidase were validated by Cell Signaling via CST western immunoblotting. In our experiments, we validated the anitibodies by performing Western blots and immunohistochemistry on cells not transfected with an EGFP or EBFP cDNA.

Eukaryotic cell lines

Policy information about cell lines and Sex and Gender in Research

Cell line source(s)	CHO-K1 cells were purchased from Millipore Sigma.
Authentication	Cells were authenticated by Millipore Sigma prior to purchase.
Mycoplasma contamination	All cells tested negative for mycoplasma contamination.
Commonly misidentified lines<br>(See ICLAC register)	*Name any commonly misidentified cell lines used in the study and provide a rationale for their use.*
